# Assessment of azithromycin-induced toxicity in *Caenorhabditis elegans*: Effects on morphology, behavior, and lipid metabolism

**DOI:** 10.1016/j.toxrep.2024.101832

**Published:** 2024-11-28

**Authors:** Elisa Kalugendo, Aamir Nazir, Rakhi Agarwal

**Affiliations:** a Laboratory of Forensic Chemistry and Toxicology, School of Forensic Sciences, National Forensic Sciences University, Delhi, India; b Division of Toxicology and Experimental Medicine, CSIR-Central Drug Research Institute, Lucknow, Uttar Pradesh, India

**Keywords:** *C. elegans*, Antibiotics, Behavior toxicity, Environmental pollution, Reproductive toxicity, Azithromycin

## Abstract

Antibiotics are indispensable in modern healthcare, playing a critical role in mitigating bacterial infections. Azithromycin is used to fight upper respiratory tract infections, however has potential toxic effects that remain inadequately understood. In our present study, azithromycin exposure to *Caenorhabditis elegans* led to significant physiological and behavioral change, with pronounced effects observed at the studied concentration. The study employs an N2 wild-type strain to examine key physiological and behavioral parameters within the worm. *C.elegans* were exposed to two concentrations of azithromycin (0.0038 and 0.00038 mg/ml) from the embryonic stage to the L4 stage for 48 hours. The study assessed key endpoints including body length, thrashing behavior, brood size, embryonic viability, lipid accumulation via Nile red staining, pharyngeal pumping rate, and response to 1-Nonanol (which assesses neurotransmitter function). Results showed that at 0.0038 mg/ml, azithromycin significantly reduced body length, increased progeny production, altered lipid deposition, delayed response to 1-Nonanol, and decreased feeding rates. Even at the lowest concentration (0.00038 mg/ml), changes in body length and lipid accumulation were observed. These findings suggest that the toxicity of azithromycin in *C.elegans* is dose-dependent and varies with exposure duration and developmental stage. Further research is needed to elucidate the molecular mechanisms underlying these toxic effects, particularly at environmentally relevant concentrations of azithromycin.

## Highlights

• At low doses, azithromycin (AZM), has potential toxicity.
• Azithromycin-induced behavioral toxicity.
• The body morphology of *C. elegans* was altered by Azithromycin.
• The feeding and thrashing activities of *C. elegan*s were inhibited at the highest concentrations of Azithromycin.
• Azithromycin stimulated the lipid content deposition, brood size, and embryonic viability of *C. elegans* at the highest concentration.

## 1 Introduction

Ecotoxicologists worldwide are working hard to evaluate the toxicological risk of pharmaceuticals on aquatic organisms and humans. It is projected that antibiotic consumption in 2030 maybe 200 % more than the 42 billion specified daily doses that were projected in 2015 [61]. Antibiotics often undergo incomplete metabolism after administration, with a substantial amount of portion being excreted in their original form in a stool or urine, which eventually enters into the sewage systems and gets integrated into the aquatic ecosystem [18]. Moreover, several antibiotics still lack adequate ecotoxicological evidence [87] including azithromycin, measured at comparatively high concentrations in aquatic environments. Azithromycin is a broad-spectrum antibiotic used heavily in veterinary and human medicine and is renowned for its antimicrobial prowess. However, mounting concern surrounds its potential adverse effects, and toxicity profiles, including organismal-level toxicity which remain insufficiently elucidated (M.-Q. [97]). The rapid emergence of antimicrobial resistance poses a substantial global health threat, necessitating continuous scrutiny of antibiotic efficacy and safety, particularly regarding their toxicity to untargeted organisms [20].

Azithromycin (AZM) has significant implications in both clinical and environmental contexts especially for vulnerable populations like diabetic patients and untargeted organisms. In diabetic patients, azithromycin (AZM) can induce prolonged QT intervals and result in fatal arrhythmias [70]. Forensically, in suicide missions, azithromycin (AZM) when combined with other drugs like insulin results in severe hypoglycemia and organ failure hence leading to death [57]. The persistence of azithromycin in the environment matrices can pose neurotoxic effects risk to non-target organisms, potentially disrupting their nervous system [59]. Untargeted or non-targeted organisms like fish, invertebrates and plants that are unintentionally exposed to pollutants such as antibiotics, chemicals, or pesticides in the environment and suffer unintended effects due to this exposure [32]. Potential environmental toxicities of antibacterial agents, such as sulfonamides, macrolides, and fluoroquinolones, hampered snails, water fleas, duckweed, cyanobacteria, and photo bacteria from growing, moving, and surviving ([41]; N. S. [81]). Antibiotics cause deformity and change in immunological responses and impede development in Walleye and Carp [26], [91]. Different studies have demonstrated the ecotoxicity of different pharmaceuticals including antibiotics exhibit pseudo-persistence in the aquatic environment due to their slow degradation and long half-life ([33]; L. [49]).

Antibiotic concentrations in surface water are not strictly regulated by environmental agencies globally, but instead, their ecological risk assessment data suggest its limits based on ecotoxicity data [5], [90]. A study conducted in Ukraine reported 0.03 mg/ml of azithromycin in surface water [92]. We have conducted monitoring studies in Sabarmati River, Gujarat, India, and identified a maximum concentration of 0.00038 mg/ml of azithromycin (Unpublished study). Another study conducted by [72] on wastewater going to the rivers in Portugal around 1.5773 µg/l of azithromycin was detected. In another study, macrolides were detected at 1.931 µg/l [59] in surface water, and 25 µg/l of azithromycin was detected in a wastewater treatment plant [76]. In some regions, concentrations of antibiotics have been observed in the (0.1 – 1) µg/L range, with no or minimum risk to aquatic organisms, particularly to primary producers like algae and other organisms in the high trophic levels in the ecosystem [52], [77]. Researchers have explored the predicted no-effect concentration of azithromycin was 0.09 µg/l in fish, 120 µg/l in *Daphnia magna,* and 0.019 µg/l in algae [6], [86]. Using immobilization assay, it was found that 48 hours of exposure to azithromycin in invertebrates including crustaceans, and *Daphnia magna* displayed the EC_**50**_ of 120,000 µg/l for acute toxicity [12], [27]. Chronic toxicity for 7-day exposure showed 4.4 µg/l had no observed effects on invertebrate reproduction and other physiological patterns [66].

Azithromycin is extensively used to treat respiratory infections, but its potentially toxic effects in the environment on living organisms are insufficiently studied and reported. The study aims to address this gap by understanding azithromycin's potentially toxic effects on non-targeted organisms as its environmental presence grows. Using the *C.elegans* model, this research examines how azithromycin impacts physiology and behavior of the animal across environmentally relevant concentrations. In the current study, *C.elegans* was subjected to azithromycin at concentrations relevant to environmental conditions. The study explored the potential impacts of azithromycin on feeding & locomotion habits, body morphology, reproductive processes, lipids deposition, and response towards the 1-Nononal compound. Several benefits have led to widespread usage of *C.elegans* as a model organism, including its short life cycle, ease of maintenance, suitability for use in a laboratory setting, and a well-characterized genome [88]. Utilizing *C.elegans* for toxicity screening provides high-throughput capabilities, facilitating sophisticated assessment of behavioral toxicity and insights into antibiotic neurotoxic, reproductive, and genotoxic effects, along with the underlying mechanisms driving these changes.

## 2 Materials and methods

### 2.1 Chemicals and reagents

Azithromycin (AZM, (CAS:21187–98–4) was purchased from Sigma Aldrich, USA. The antibiotics were dissolved in pure dimethyl sulfoxide (DMSO) (Merck Millipore, Mumbai India) followed by dilution with a specified solvent, 1 % DMSO was used in the total solution as previously described (S. [50]). Sodium chloride, magnesium sulfate, cholesterol, peptone, agar, potassium dihydrogen phosphate, uracil, dextrose, sodium hydroxide, sodium hypochlorite solution, sodium azide, and sodium hydrogen phosphate, were procured from SRL Pvt. Ltd. Merck Millipore, Mumbai India and all of the chemicals were of analytical grade with 99 % purity. The stock concentration of azithromycin was 0.38 mg/ml and was maintained in the dark at 4°C before usage.

### 2.2 *C. elegans* and *E.coli* culture

The nematode strains were sourced from the *Caenorhabditis elegans* Genetics Center at the University of Minnesota, USA. N2 strains of *C. elegans* were grown on nematode growth media, with *E. coli* OP50 serving as the nematode's food source, and incubated at 22°C. Adult gravid worms were subjected to sodium hypochlorite treatment to acquire age-synchronized embryos, which were then cultured on a new NGM plate seeded with OP50. To make 100 ml of *E.coli* 100 ml MEM was inoculated with 1 ml of *E.coli* stock culture and incubated overnight at 37°C at 180 rpm. After incubation, the OP50 culture was kept at 4°C in 50 ml falcon tubes as described by [36].

### 2.3 Exposure conditions to azithromycin

The final working concentration used was 0.0038 mg/ml and 0.00038 mg/ml of azithromycin. The subsequent antibiotic was mixed with OP50 and seeded with the Nematode Growth Media (NGM) plates. With continuous exposure, the embryos were suspended on the antibiotic-seeded plates and exposed for 48 hours up to the L-4 stage.

### 2.4 Body morphology alteration

The NGM plates were seeded with OP50 bacteria and varying concentrations of azithromycin to investigate the dose response of these antibiotics in *C. elegans.* The plates were then incubated at 22°C for 12 hours. The following day, each group was inoculated with 200 µL of embryos, covered with parafilm, and kept at 22°C for 48 hours. Subsequently, L4 stage worms were collected and thoroughly washed with M-9 buffer to eliminate bacteria and debris. To immobilize the worms, 20 µL of sodium azide was added to each group. Then, 20 µL of nematodes were transferred onto slides and covered with a cover slip. Using a fluorescence microscope at 10x magnification, 15 images of worms from each group were captured.The body length of these 15 nematodes in each group was then analyzed using Image J software with freehand line tools (Z. [95]).

### 2.5 Thrashing assay

The embryos were placed on NGM plates seeded with azithromycin OP50 and DMSO, and incubated for 48 hours. After collecting L-4 stage worms and thoroughly cleaning them with M-9 buffer, 20 µl of the worms which included between (20−30) worms were added to the NGM plates without any food and given a brief period to move freely and acclimatize to their new environment. Next, we took a 30-second video using Leica software with the aid of a stereo zoom microscope (Leica EZ4D) for each group, during which we counted the number of thrashes in 10 seconds and examined at least 20 worms per group as explained elsewhere by [23].

### 2.6 Body bending behavior

The embryos were placed on NGM plates seeded with azithromycin, OP50, and DMSO, and incubated for 48 hours. After collecting L-4 stage worms and thoroughly cleaning them with M-9 buffer, 5 µl of them containing (5−11) worms were added to the freshly unseeded NGM plates and given enough time to acclimatize with their new environment. We took a 30-second video using Leica software with the aid of a stereo zoom microscope (Leica EZ4D) for each group, during which we counted the number of body bending in 10 seconds and examined at least 20 worms per group as explained by [60].

### 2.7 1-Nonanol assay

The effects of azithromycin and a control group on NGM plates were examined for evidence of antibiotic-induced neurotransmitter impairment. 1-Nnonal is used to test dopamine signaling in *C.elegans* by triggering avoidance behavior, changes in this response indicate a potential neurotoxic effect. In this investigation, we soaked the worm picker in 200 µl of 1-Nonanol. The worm picker was then positioned close to the snout of a moving active worm under a stereo microscope, and we timed how long it took the worm to get away from the 1-Nonanol chemical, at least 20 worms were used from each group as detailed by [80].

### 2.8 Pharyngeal pumping

Pharyngeal pumping serves as a method for measuring the food intake by worms and was performed as explained by [42]. Briefly L-4 stage worms, after being rinsed with M-9 buffer, were placed into microcentrifuge tubes. 20 µl of nematode solution was added to freshly seeded plates. Nematodes were allowed 30 minutes to adjust to their new environment, during which they moved freely and began feeding. Pharyngeal pumping activity was recorded for 10 seconds at an 8x magnification using a stereo zoom microscope. The feeding behavior of a minimum of 20 worms in each group was recorded.

### 2.9 Lipid content estimation

Nile red staining was employed to assess the lipid levels in N2 wild-type worms subjected to treatment with azithromycin. To prepare the Nile red solution, 0.5 mg of the dye was dissolved in 1 ml of acetone and stored at −20°C in darkness. The working solution was created by mixing 3 µL of the dye with 750 µL of OP50 for control samples and then adjusting the final volume with compounds. The next day embryos were seeded in the plates, after 48 hours, L4 worms from each group were collected, thoroughly washed to remove bacteria, and treated with 20 µl of sodium azide. Subsequently, 20 µl of the worms were placed on a slide and covered with a coverslip. Nematode images were captured using fluorescence microscopy with a rhodamine filter at 20x magnification. Image J software was utilized to analyze at least 15 images per group, enabling quantification of lipid content in *C.elegans* based on the fluorescence intensity of Nile red as explained elsewhere by [83].

### 2.10 Brood size & reproductive age

Ten L4-stage age-synchronized worms were moved to freshly prepared plates from each experimental group. After 24 hours, the number of embryos and L1-stage progenies was counted following the careful transfer of worms to newly prepared plates, ensuring no embryos or L-1 worms were transferred. Worms were transferred every 24 hours until they ceased producing embryos, and an average brood size was calculated. To assess the reproductive age in *C.elegans* treated with azithromycin, the group's average time for the worms to cease producing eggs was recorded as described by [43].

### 2.11 Embryonic viability

Following the transfer of L-4 stage worms to newly seeded plates, the progenies counting was conducted the following day. These plates were then left undisturbed for 24 hours to ensure sufficient time for all embryos on the plate to hatch. The number of unhatched embryos and live progenies was noted, and the experiment proceeded similarly for all groups until no embryos were laid by the worms. Subsequently, the embryonic viability in each group was calculated according to [44]**.**

### 2.12 Statistical analysis

The statistical significance of the obtained data was assessed using a Student t-test through GraphPad Prism 5, with a significance level set at (P<0.05), (P<0.005), P<0.0001), and (P<0.0005). The non-parametric independent student t-test was implemented and the P-values adjustment was adopted for post-multiple comparisons. All experiments included a minimum of two separate experimental trials, the result data were represented graphically with error bars indicating the minimum standard error of the mean.

## 3 Results

### 3.1 Body morphology alteration

**Fig. 1:**
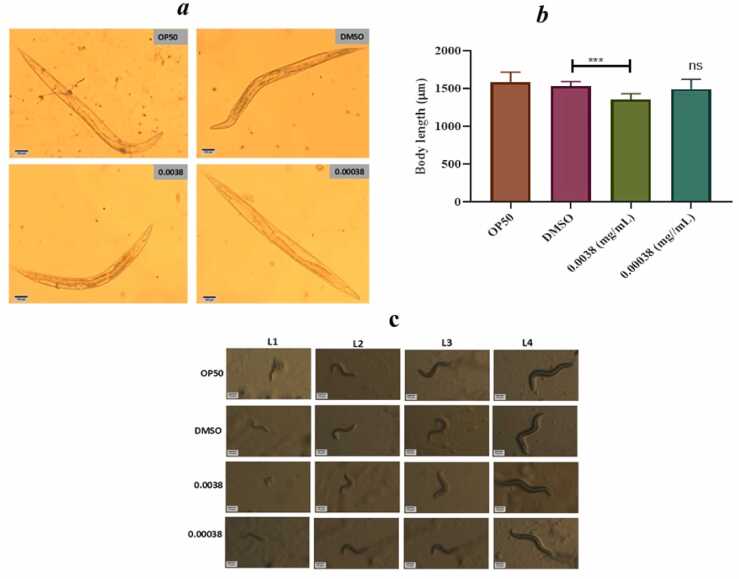
(a-b) shows the highest concentration of azithromycin shortened the body length of *C.elegans* and (Fig. 1c) shows how the maximal dose of azithromycin impacted the growth and developmental progression in *C. elegans* as quantified by non-parametric independent t-test. Error bars indicate the standard error of the mean, ( ****p<0.0005) and ns-non significant.

The impact of azithromycin on the development and growth of *C.elegans* is displayed in Fig. 1**(a-b)**. It was found that at the highest concentration of azithromycin, the body length of *C.elegans* was reduced and the nematode could not reach its full body length at the L-4 stage when compared to the control groups. In another case, there was a delay of a nematode to transform from one stage to another at the highest concentration of azithromycin as seen in Fig. 1**c.**

### 3.2 Thrashing & body bending behavior influenced by azithromycin in *C. elegans*

**Fig. 2:**
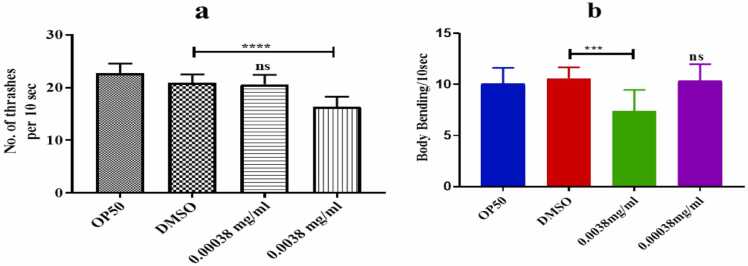
(a-b) shows that azithromycin affected the head thrashing and body bending behavior of *C. elegans* as quantified by a non-parametric independent t-test. Error bars indicate the standard error of the mean), (***p<0.0005), (****p<0.0001), and ns-non significant.

At the highest concentration of 0.0038 mg/ml, azithromycin significantly slowed down the head thrashing and body bending capability of *C.elegans* as seen in Fig. 2a and refer to video-1. *C.elegans* exhibited a significant decrease in head thrashing body and bending frequencies at the highest concentration when compared to the control group, while there was no significant difference in body bending frequencies at the lowest concentration. This suggests that the azithromycin impact on locomotion behavior in *C.elegans* is dose-dependent.

Supplementary material related to this article can be found online at doi:10.1016/j.toxrep.2024.101832.

The following is the Supplementary material related to this article Video S1, Video S2.

Video S1

Video S2

### 3.3 1-Nonanol response influenced by azithromycin in *C. elegans*

**Fig. 3:**
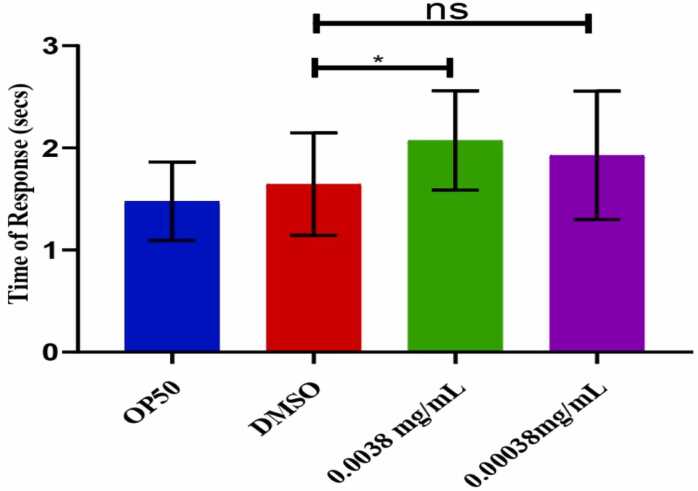
above shows the response of *C.elegans* towards the 1-Nonanol compound as quantified by a non-parametric independent t-test. Error bars indicate the standard error of the mean), (*p<0.05), and ns-non significant.

The scenario depicted in Fig. 3 above demonstrates a notable impact on *C.elegans* chemoreceptors and neurotransmission. Upon exposure to the chemical 1-Nonanol, the nematodes exhibited a delayed response in moving away from the chemical stimuli.

### 3.4 Feeding behavior of *C. elegans* when treated with azithromycin

**Fig. 4:**
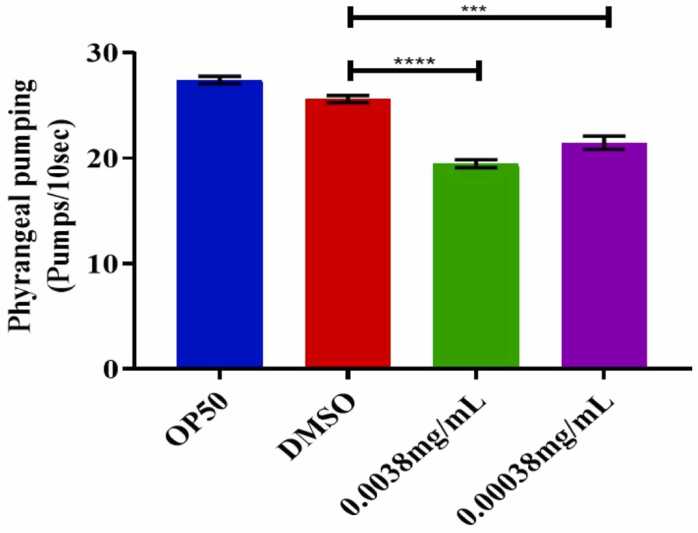
above shows that at both high and low doses, azithromycin inhibited the feeding behavior of *C. elegans* as quantified by a non-parametric independent t-test. Error bars indicate the standard error of the mean, ***p<0.0001, ****p<0.0005, and ns-non significant.

The pharyngeal pumping was significantly decreased in nematodes at both the highest and lowest concentration of azithromycin as depicted in Fig. 4 and by referring to supplementary **video-2**. This might have been characterized by nematode-reduced capacity for food intake and may have resulted from azithromycin-induced neuromuscular disturbance and mitochondrial dysfunction.

### 3.5 The impact of azithromycin on lipid deposition in *C. elegans*

**Fig. 5:**
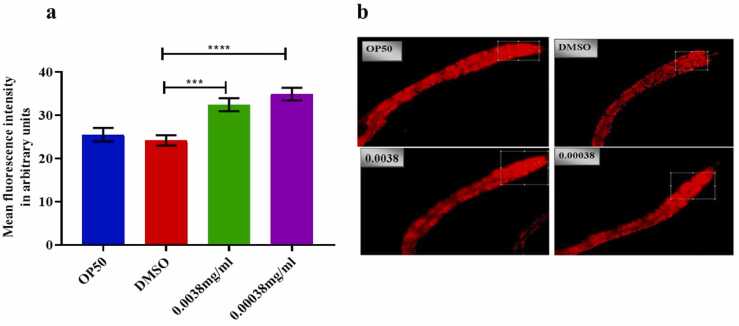
**(a-b**) shows that azithromycin influenced the increase of lipid deposition in N2 *C. elegans* at both low and high dosages as quantified by a non-parametric independent t-test. Error bars indicate the standard error of the mean, (***p<0.0001), (****p<0.0005), and ns-non significant.

As depicted in Fig. 5**(a-b)** above**,** it was found that azithromycin influenced the increase in lipid content at both the highest and lowest concentrations respectively (0.0038–0.00038) mg/ml.

### 3.6 The impact of azithromycin on reproductive health in *C. elegans*

**Fig. 6:**
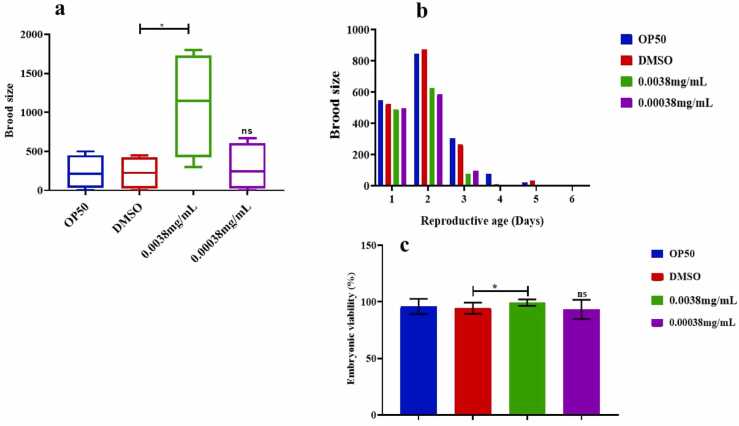
(a) shows that azithromycin stimulated the increase of brood size in *C. elegans* at its highest concentration, Fig. 6(b) shows that reproductive age was shortened at the lowest concentration, and Fig. 6c above shows that there was no significant difference in embryonic viability at the lowest concentration of azithromycin in *C.elegans* as quantified by non-parametric independent t-test. Error bars indicate the standard error of the mean and ns-non significant.

It is apparent that at a concentration of 0.0038 mg/ml, azithromycin stimulated the reproductive potential in *C.elegans* as seen in the number of progenies is higher compared to the lowest concentration. As displayed in Fig. 6**(a-b)** the reproductive days increased in the treatment group at the highest concentration but declined at the lowest concentration of azithromycin. In another case, azithromycin significantly stimulated the embryonic viability in *C. elegans* at the highest concentration but was non-significant at the lowest concentration of 0.00038 mg/ml as portrayed in Fig. 6**c.**

## 4 Discussion

Azithromycin, a broad-spectrum macrolide, is one of the most frequently prescribed antibiotics due to its high stability in acidic conditions, longer serum half-life, and its ability to achieve higher concentrations in animal tissues compared to erythromycin, to which it is structurally related. These properties contribute to its environmental persistence, making it a significant environmental risk [2]. Azithromycin gained significant attention during the COVID-19 pandemic and is often used in combination with drugs like hydroxychloroquine [24]. Its widespread during the pandemic led to increased environmental discharge and subsequent risk to the aquatic system [40], [9]. Despite the absence of specific regulations on surface water levels for antibiotics, azithromycin is included in the European Water Framework Directive's "watch list" due to its toxicity, persistence, and bio-accumulative potential [17].

In the current study, the resulting reduction of pharyngeal pumping in *C.elegans* by azithromycin might be due to the disruption of gut microbiota and energy depletion causing mitochondrial dysfunction and induction of neuromuscular toxicity [8]. However with other antibiotics like sulfamethoxazole, (S. [51]) have discovered that *C.elegans* showed a rise in pharyngeal pumping, which was not the case when *C.elegans* were subjected to azithromycin. Furthermore, [10] obtained similar results when Zebrafish were subjected to azithromycin leading to a reduced food intake and vascular irregularities. These findings may provide an early indication of the potential adverse effects of azithromycin in humans, especially its impact on gastrointestinal motility [11] and neuromuscular functioning. Azithromycin may have adverse effects in humans such as gastrointestinal symptoms including nausea, vomiting, abdominal pain, and diarrhea [45]. These symptoms are directly related to azithromycin's influence on the enteric nervous system and smooth muscle functioning. Ruszkiewicz et al., [73]**.** Moreover neuromuscular disorders like myasthenia gravis [78], muscle weakness and fatigue are insinuated by azithromycin through neuromuscular exacerbation has been reported [67].

Furthermore, due to poor food intake, the body morphology and overall growth of *C.elegans* were affected and led to the retardation of growth. It is reported that azithromycin severely impairs mitochondrial DNA and enzymes like DNA gyrase and topoisomerase IV which are crucial for DNA replication and compromise the process of cell division and cellular processes that are essential for growth and development [71]. Erythromycin has been shown in earlier studies to affect body width and length, with a modest inhibition of body length observed at 1.0 μg/L (Z. [47]). In other studies, azithromycin exposed to anuran amphibian larvae resulted in a declined body size and shape due to the loss of the ability to feed the animals becoming weak, and thin and inducing liver toxicity in Zebrafish [13], [63]. Azithromycin may block the cellular processes in humans particularly actively dividing cells, which are crucial for growth and development in children, pregnant women, and patients with healing tissues (Z. [39]). Furthermore, azithromycin impairs protein synthesis and slows down growth and development [28], [53], especially in growing children. It has been reported that the impact on gastrointestinal function leads to poor nutrient absorption, which relates to poor growth, and weight loss which triggers inflammations in the gastrointestinal and results in conditions like colitis [62]. This may further impairment of nutrient absorption and tissue repair [84]. During pregnancy, the risk of developmental toxicity from azithromycin may lead to congenital anomalies like low birth weight or even miscarriage [4].

Body bending is the crawling behavior of worms in which *c.elegans* bends its head region across the central line of the body and forms an alternate longitudinal crest behind the pharynx followed by a longitudinal trough which completes 1 body bend [65]. *C.elegans* has 302 neurons, 6393 synapses, and several neurotransmitters like acetylcholine, dopamine, serotonin, GABA, tyramine, and octopamine [30]. Out of these neurotransmitters, acetylcholine helps in muscle contraction and helps in locomotion [74]. The highest concentrations of azithromycin might have induced acetylcholinesterase inhibitory activity thus leading to low body bending frequencies in *C.elegans.* The observed decrease in response to 1-nonanol compound, head thrashing and body bending behavior frequencies in *C.elegans* at the highest concentrations of azithromycin. This may suggest a neurotoxic or motor impairment, which can be loosely related to how certain drugs impact human motor functioning.

Moreover, the thrashing activity and response towards chemical stimuli in *C.elegans* were altered probably due to the reduction in food intake the animals became weak and thin. Locomotion activities slowed down and their response toward the 1-Nononal compound was prolonged [64], [79]. Due to hampering the functionality of ATP-dependent channels and pumps led to compromised muscle contraction. Azithromycin causes damage to the G-protein coupled receptors, membrane proteins, and ion channels disrupting cyclic AMP (cAMP) and calcium signaling pathways, crucial for the execution of sensory perception and avoidance behavior [89]. Pharmaceutical compound screening by using *C.elegans* [22], [68], pointed out that antibiotics affected the locomotion behavior and impaired chemosensory receptors. Chicks and Quills were treated with 7305 mg/kg and 11.169 mg/kg respectively of azithromycin and there was a notable decrease in their movement due to the effects of their neurobehavior and motor measure [1]. Azithromycin has the potential to cause muscle toxicity like rhabdomyolysis [21] which relates to serious muscle pain, weakness, and kidney failure [35]. Neurological side effects like dizziness, confusion, and impaired motor function have been observed in elderly patients [56]. Compromised reflexes and coordination can block daily functioning and accelerate accident risks to vulnerable populations [25]. A pediatric patient administered 500 mg/d of azithromycin developed agitation and choreoathetosis movements on the third day of administration [19]. Chemosensory side effects of antibiotics result from disruption of transduction pathways, biochemical targets, and enzymes [75]. Adverse effects like anorexia have been observed during azithromycin administration which induces taste and smell disorders [34].

Along with the behavioral changes, lipid metabolism was altered when *C.elegans* were exposed to azithromycin. Stresses from reactive oxygen species thus activated mitogen-activated kinase pathways may contribute to the upregulation of fatty acid synthase and acetyl-CoA carboxylase genes which are involved in lipogenesis [7]. Reduced β-Oxidation of fatty acids by antibiotics promoted fatty storage in *C.elegans* [3]. Furthermore, the impairment of the Electron Transport Chain (ETC) and decrease in ATP production compromised the availability of NADH and FADH2 hence poor fatty acid oxidation and led to the suppression of anabolic processes [58]. Antibiotics were found to encourage obesity in *C.elegans* (Z. [94]). Moreover, sulfonamides in *Daphnia magna* revealed inhibition of lipase and acetylcholinesterase enzyme activities crucial in lipid metabolism (Y. [98]), this led to the accumulation of fats, increased body size, and elevated levels of triglycerides in the organism (Z. [48]). Azithromycin can disrupt lipid metabolism in humans which can result in dyslipidemia ((L. [38])). Characterized by abnormal blood lipid levels that can increase the risk of cardiac failure [85]. Furthermore, it could elevate cholesterol and fat accumulation, leading to obesity (J. [93]). Disruption in lipid metabolism can alter energy balance leading to weight gain and obesity and increasing the risk of atherosclerosis [29]. Impaired lipid signaling and insulin sensitivity can contribute to metabolic syndrome, hypertension, and type 2 diabetes [96].

Perhaps balanced lipid metabolism is essential for reproductive health in *C.elegans* by providing essential energy for oocyte development and embryogenesis. Excessive lipid accumulation may disrupt insulin/Insulin-like Growth factor (IGF-1) signaling and activated protein kinase (AMPK) leading to accelerated reproductive aging and impaired oocyte quality [69]. Antibiotics decreased the brood size, number of fertilized eggs in the uterus, and reduced the number of total germline cells, and ovulation rate in *C. elegans* [99]*.* Macrolides registered high toxicity by causing progressive impairment in reproduction and induced a high mortality rate in *Daphnia magna* and microalgae over multiple generations [14], [55]. Contrary erythromycin significantly inhibited reproduction across multiple generations and consistently suppressed fatty acid synthase, and the same effects were observed in *D.melanogaster* (Z. [48], [54]). In humans, azithromycin could interfere with the hormonal system and potentially lead to fertility issues, menstrual irregularities, and pregnancy complications (Y. [46]). Hormonal imbalances may cause conditions like polycystic ovary syndrome, affecting hormone levels and fertility ([31]; S. [82]). This can lead to developmental issues in embryos and increase the risk of birth defects [16]. Clinically, azithromycin's effects on reproductive organs may lead to conditions such as testicular and ovarian dysfunction and reduced sperm quality (F. Y. S. [37]). Azithromycin should be administered with precaution during pregnancy because azithromycin can result in teratogenic effects on a developing fetus [15].

## 5 Conclusion

This study reveals that azithromycin exhibits dose-dependent, exposure duration, and developmental stage to induce toxicological effects on *C.elegans* by affecting feeding, growth, locomotion, lipid metabolism, and reproduction. This toxicity underscores the forensic significance of azithromycin whereby clinical toxicologists, ecotoxicologists, and environmental protection pioneers may use this data to assess ecological contamination, investigate cases of human poisoning, influence regulation and legal actions related to public health and environmental safety by establishing standardized protocols limiting antibiotic levels in the environment. Additional research into the genetic and proteomic toxicological impacts of antibiotics at their relevant environmental concentration is highly recommended. Also, there is a critical need for studies that assess the toxicity of antibiotics in combination with other aquatic pollutants like heavy metals along with the wise and safe use of this drug.

## Funding

No financial support was provided for this research.

## CRediT authorship contribution statement

**Rakhi Agarwal:** Visualization, Supervision, Resources, Project administration, Conceptualization. **Elisa Kalugendo:** Writing – original draft, Software, Methodology, Investigation, Formal analysis. **Aamir Nazir:** Writing – review & editing, Validation, Supervision, Resources, Data curation, Conceptualization.

## Declaration of Competing Interest

The authors declare that they have no known competing financial interests or personal relationships that could have appeared to influence the work reported in this paper

## Data Availability

Data will be made available on request.

## References

[1] Al-Abdaly Y.Z., Alfathi M.Y., Al-mahmood S.S. (2023). Comparison of azithromycin toxicity in chickens and quails. Iran. J. Vet. Med..

[2] Almeida A.C., Gomes T., Lomba J.A.B., Lillicrap A. (2021). Specific toxicity of azithromycin to the freshwater microalga Raphidocelis subcapitata. Ecotoxicol. Environ. Saf..

[3] An L., Fu X., Chen J., Ma J. (2023). Application of caenorhabditis elegans in lipid metabolism research. Int. J. Mol. Sci..

[4] Antonucci R., Cuzzolin L., Locci C., Dessole F., Capobianco G. (2022). Use of azithromycin in pregnancy: more doubts than certainties. Clin. Drug Investig..

[5] Aus Der Beek T., Weber F., Bergmann A., Hickmann S., Ebert I., Hein A., Küster A. (2016). Pharmaceuticals in the environment—Global occurrences and perspectives. Environ. Toxicol. Chem..

[6] Aydin S., Aydin M.E., Ulvi A., Kilic H. (2019). Antibiotics in hospital effluents: occurrence, contribution to urban wastewater, removal in a wastewater treatment plant, and environmental risk assessment. Environ. Sci. Pollut. Res..

[7] Batchuluun B., Pinkosky S.L., Steinberg G.R. (2022). Lipogenesis inhibitors: therapeutic opportunities and challenges. Nat. Rev. Drug Discov..

[8] Bonuccelli G., Brooks D.R., Shepherd S., Sotgia F., Lisanti M.P. (2023). Antibiotics that target mitochondria extend lifespan in C. elegans. Aging.

[9] Butler C.C., Dorward J., Yu L.-M., Gbinigie O., Hayward G., Saville B.R., Van Hecke O., Berry N., Detry M., Saunders C., Fitzgerald M., Harris V., Patel M.G., De Lusignan S., Ogburn E., Evans P.H., Thomas N.P., Hobbs F.R. (2021). Azithromycin for community treatment of suspected COVID-19 in people at increased risk of an adverse clinical course in the UK (PRINCIPLE): A randomised, controlled, open-label, adaptive platform trial. Lancet.

[10] Chen C., Song J., Pu Q., Liu X., Yan J., Wang X., Wang H., Qian Q. (2023). Azithromycin induces neurotoxicity in zebrafish by interfering with the VEGF/Notch signaling pathway. Sci. Total Environ..

[11] Chini P., Toskes P.P., Waseem S., Hou W., McDonald R., Moshiree B. (2012). Effect of azithromycin on small bowel motility in patients with gastrointestinal dysmotility. Scand. J. Gastroenterol..

[12] Cunningham V.L., Buzby M., Hutchinson T., Mastrocco F., Parke N., Roden N. (2006). Effects of human pharmaceuticals on aquatic life: next steps. Environ. Sci. Technol..

[13] Dall K.B., Færgeman N.J. (2019). Metabolic regulation of lifespan from a C. elegans perspective. Genes Nutr..

[14] Dalla Bona M., Lizzi F., Borgato A., De Liguoro M. (2016). Increasing toxicity of enrofloxacin over four generations of Daphnia magna. Ecotoxicol. Environ. Saf..

[15] Danielsson B.R., Barrow P.C. (2013).

[16] De Felici M., Klinger F.G., Farini D., Scaldaferri M.L., Iona S., Lobascio M. (2005). Establishment of oocyte population in the fetal ovary: primordial germ cell proliferation and oocyte programmed cell death. Reprod. Biomed. Online.

[17] Efrain Merma Chacca D., Maldonado I., Vilca F.Z. (2022). Environmental and ecotoxicological effects of drugs used for the treatment of COVID 19. Front. Environ. Sci..

[18] European Environment Agency. (2010). *Pharmaceuticals in the environment: Results of an EEA workshop*. Publications Office. 〈https://data.europa.eu/doi/10.2800/31181〉.

[19] Farooq O., Memon Z., Stojanovski S.D., Faden H.S. (2011). Azithromycin-induced agitation and choreoathetosis. Pediatr. Neurol..

[20] Fernandes M.J., Paíga P., Silva A., Llaguno C.P., Carvalho M., Vázquez F.M., Delerue-Matos C. (2020). Antibiotics and antidepressants occurrence in surface waters and sediments collected in the north of Portugal. Chemosphere.

[21] Finsterer J., Stollberger C.C., Melichart-Kotig M. (2022). Rhabdomyolysis triggered by azithromycin. J. Fam. Med. Prim. Care.

[22] Fryer E., Guha S., Rogel-Hernandez L.E., Logan-Garbisch T., Farah H., Rezaei E., Mollhoff I.N., Nekimken A.L., Xu A., Fechner S., Druckmann S., Clandinin T.R., Rhee S.Y., Goodman M.B. (2023). An efficient behavioral screening platform classifies natural products and other chemical cues according to their chemosensory valence in C. elegans [Preprint]. Neuroscience.

[23] Gaur A.V., Agarwal R. (2021). Risperidone induced alterations in feeding and locomotion behavior of Caenorhabditis elegans. Curr. Res. Toxicol..

[24] Gautret P., Lagier J.-C., Parola P., Hoang V.T., Meddeb L., Mailhe M., Doudier B., Courjon J., Giordanengo V., Vieira V.E., Tissot Dupont H., Honoré S., Colson P., Chabrière E., La Scola B., Rolain J.-M., Brouqui P., Raoult D. (2020). Hydroxychloroquine and azithromycin as a treatment of COVID-19: Results of an open-label non-randomized clinical trial. Int. J. Antimicrob. Agents.

[25] Grill M.F., Maganti R.K. (2011). Neurotoxic effects associated with antibiotic use: management considerations. Br. J. Clin. Pharmacol..

[26] Hanna N., Sun P., Sun Q., Li X., Yang X., Ji X., Zou H., Ottoson J., Nilsson L.E., Berglund B., Dyar O.J., Tamhankar A.J., Stålsby Lundborg C. (2018). Presence of antibiotic residues in various environmental compartments of Shandong province in eastern China: Its potential for resistance development and ecological and human risk. Environ. Int..

[27] Harada A., Komori K., Nakada N., Kitamura K., Suzuki Y. (2008). Biological effects of PPCPs on aquatic lives and evaluation of river waters affected by different wastewater treatment levels. Water Sci. Technol..

[28] Heidary M., Ebrahimi Samangani A., Kargari A., Kiani Nejad A., Yashmi I., Motahar M., Taki E., Khoshnood S. (2022). Mechanism of action, resistance, synergism, and clinical implications of azithromycin. J. Clin. Lab. Anal..

[29] Henning, R.J. (2021). *Obesity and obesity-induced inflammatory disease contribute to atherosclerosis: A review of the pathophysiology and treatment of obesity*. PMC8449192 34548951

[30] Hobert, O. (2018). The neuronal genome of Caenorhabditis elegans. In *WormBook: The Online Review of C. elegans Biology [Internet]*. WormBook. 〈https://www.ncbi.nlm.nih.gov/books/NBK154158/〉. 10.1895/wormbook.1.161.1 PMC4781646 24081909

[31] Hou L., Fu Y., Zhao C., Fan L., Hu H., Yin S. (2024). The research progress on the impact of antibiotics on the male reproductive system. Environ. Int..

[32] Impellitteri F., Multisanti C.R., Rusanova P., Piccione G., Falco F., Faggio C. (2023). Exploring the impact of contaminants of emerging concern on fish and invertebrates physiology in the mediterranean sea. Biology.

[33] Jani K., Dhotre D., Bandal J., Shouche Y., Suryavanshi M., Rale V., Sharma A. (2018). World’s largest mass bathing event influences the bacterial communities of godavari, a holy river of India. Microb. Ecol..

[34] Kan Y., Nagai J., Uesawa Y. (2021). Evaluation of antibiotic-induced taste and smell disorders using the FDA adverse event reporting system database. Sci. Rep..

[35] Kato K., Iwasaki Y., Onodera K., Higuchi M., Kato K., Kato Y., Tsutsui M., Taniguchi M., Furukawa H. (2016). Pregabalin- and azithromycin-induced rhabdomyolysis with purpura: an unrecognized interaction: a case report. Int. J. Surg. Case Rep..

[36] Ke T., Santamaría A., Tinkov A.A., Bornhorst J., Aschner M. (2020). Generating Bacterial Foods in Toxicology Studies with *Caenorhabditis elegans*. Curr. Protoc. Toxicol..

[37] Kong F.Y.S., Rupasinghe T.W., Simpson J.A., Vodstrcil L.A., Fairley C.K., McConville M.J., Hocking J.S. (2017). Pharmacokinetics of a single 1g dose of azithromycin in rectal tissue in men. PLOS ONE.

[38] Kong L., Yu S., Gu L., Geng M., Zhang D., Cao H., Liu A., Wang Q., Wang S., Tao F., Liu K. (2022). Associations of typical antibiotic residues with elderly blood lipids and dyslipidemia in West Anhui, China. Ecotoxicol. Environ. Saf..

[39] Kong Z., Zhu L., Liu Y., Liu Y., Chen G., Jiang T., Wang H. (2024). Effects of azithromycin exposure during pregnancy at different stages, doses and courses on testicular development in fetal mice. Biomed. Pharmacother..

[40] Kournoutou G.G., Dinos G. (2022). Azithromycin through the lens of the COVID-19 treatment. Antibiotics.

[41] Kovalakova P., Cizmas L., McDonald T.J., Marsalek B., Feng M., Sharma V.K. (2020). Occurrence and toxicity of antibiotics in the aquatic environment: a review. Chemosphere.

[42] Kuo-Esser L., Chen R., Lawson K., Kuchinski K., Simmons N., Dominguez M., Scandura T., Vo M., Dasenbrock-Gammon E., Hagan N., Esposito H., Thompson M., Le S., Escorcia W., Wetzel H.N. (2024). Early-life caffeine exposure induces morphological changes and altered physiology in *Caenorhabditis elegans*. Biochem. Biophys. Res. Commun..

[43] Kwah J.K., Jaramillo-Lambert A. (2023). Measuring embryonic viability and brood size in caenorhabditis elegans. J. Vis. Exp..

[44] Kwah J.K., Jaramillo-Lambert A. (2023). Measuring embryonic viability and brood size in caenorhabditis elegans. J. Vis. Exp..

[45] Larson J.M., Tavakkoli A., Drane W.E., Toskes P.P., Moshiree B. (2010). Advantages of azithromycin over erythromycin in improving the gastric emptying half-time in adult patients with gastroparesis. J. Neurogastroenterol. Motil..

[46] Li Y., Huang J., Ge C., Zhu S., Wang H., Zhang Y. (2024). The effects of prenatal azithromycin exposure on offspring ovarian development at different stages, doses, and courses. Biomed. Pharmacother..

[47] Li Z., Yu Z., Yin D. (2024). Influence of dietary status on the obesogenic effects of erythromycin antibiotic on Caenorhabditis elegans. Environ. Int..

[48] Li Z., Yu Z., Yin D. (2024). Influence of dietary status on the obesogenic effects of erythromycin antibiotic on *Caenorhabditis elegans*. Environ. Int..

[49] Liu L., He S., Tang M., Zhang M., Wang C., Wang Z., Sun F., Yan Y., Li H., Lin K. (2022). Pseudo toxicity abatement effect of norfloxacin and copper combined exposure on Caenorhabditis elegans. Chemosphere.

[50] Liu S., Saul N., Pan B., Menzel R., Steinberg C.E.W. (2013). The non-target organism caenorhabditis elegans withstands the impact of sulfamethoxazole. Chemosphere.

[51] Liu S., Saul N., Pan B., Menzel R., Steinberg C.E.W. (2013). The non-target organism caenorhabditis elegans withstands the impact of sulfamethoxazole. Chemosphere.

[52] Lü, D., Yu, C., Zhuo, Z., Meng, S., Liu, S., Institute of Hydrogeology and Environmental Geology, Chinese Academy of Geological Sciences, Shijiazhuang 050061, China, Key Laboratory of Groundwater Sciences and Engineering, Ministry of Natural Resources, Zhengding 050803, China, & China University of Geosciences (Beijing), Beijing 100083, China. (2022). The distribution and ecological risks of antibiotics in surface water in key cities along the lower reaches of the Yellow River: A case study of Kaifeng City, China. China Geology, 5(0), 1–10. https://doi.org/10.31035/cg2022032.

[53] Lu Z., Guo Y., Xu D., Xiao H., Dai Y., Liu K., Chen L., Wang H. (2023). Developmental toxicity and programming alterations of multiple organs in offspring induced by medication during pregnancy. Acta Pharm. Sin. B.

[54] Machado M.D., Soares E.V. (2019). Impact of erythromycin on a non-target organism: Cellular effects on the freshwater microalga Pseudokirchneriella subcapitata. Aquat. Toxicol..

[55] Mao Y., Yu Y., Ma Z., Li H., Yu W., Cao L., He Q. (2021). Azithromycin induces dual effects on microalgae: Roles of photosynthetic damage and oxidative stress. Ecotoxicol. Environ. Saf..

[56] Mattappalil A., Mergenhagen K.A. (2014). Neurotoxicity with antimicrobials in the elderly: a review. Clin. Ther..

[57] Milberg P., Eckardt L., Bruns H.-J., Biertz J., Ramtin S., Reinsch N., Fleischer D., Kirchhof P., Fabritz L., Breithardt G., Haverkamp W. (2002). Divergent proarrhythmic potential of macrolide antibiotics despite similar QT prolongation: fast phase 3 repolarization prevents early afterdepolarizations and torsade de pointes. J. Pharmacol. Exp. Ther..

[58] Miranda-Vizuete A., Veal E.A. (2017). Caenorhabditis elegans as a model for understanding ROS function in physiology and disease. Redox Biol..

[59] Mirzaie F., Teymori F., Shahcheragh S., Dobaradaran S., Arfaeinia H., Kafaei R., Sahebi S., Farjadfard S., Ramavandi B. (2022). Occurrence and distribution of azithromycin in wastewater treatment plants, seawater, and sediments of the northern part of the persian gulf around bushehr port: a comparison with Pre-COVID 19 pandemic. Chemosphere.

[60] Mishra S., Agarwal R. (2020). Assessment of behavioural toxicity in dichlorvos-exposed Caenorhabditis elegans. Environ. Exp. Biol..

[61] Pandey G K.D. (2014). Determination of drugs and metabolites in raw wastewater using liquid chromatography-mass spectrometry. J. Forensic Res..

[62] Parnham M.J., Haber V.E., Giamarellos-Bourboulis E.J., Perletti G., Verleden G.M., Vos R. (2014). Azithromycin: mechanisms of action and their relevance for clinical applications. Pharmacol. Ther..

[63] Peltzer P.M., Lajmanovich R.C., Attademo A.M., Junges C.M., Teglia C.M., Martinuzzi C., Curi L., Culzoni M.J., Goicoechea H.C. (2017). Ecotoxicity of veterinary enrofloxacin and ciprofloxacin antibiotics on anuran amphibian larvae. Environ. Toxicol. Pharmacol..

[64] Petratou D., Fragkiadaki P., Lionaki E., Tavernarakis N. (2024). Assessing locomotory rate in response to food for the identification of neuronal and muscular defects in C. elegans. STAR Protoc..

[65] Pierce-Shimomura J.T., Chen B.L., Mun J.J., Ho R., Sarkis R., McIntire S.L. (2008). Genetic analysis of crawling and swimming locomotory patterns in *C. elegans*. Proc. Natl. Acad. Sci..

[66] Pizzini S., Giubilato E., Morabito E., Barbaro E., Bonetto A., Calgaro L., Feltracco M., Semenzin E., Vecchiato M., Zangrando R., Gambaro A., Marcomini A. (2024). Contaminants of emerging concern in water and sediment of the Venice Lagoon, Italy. Environ. Res..

[67] Pradhan S., Pardasani V., Ramteke K. (2009). Azithromycin-induced myasthenic crisis: reversibility with calcium gluconate. Neurol. India.

[68] Queirós L., Marques C., Pereira J.L., Gonçalves F.J.M., Aschner M., Pereira P. (2021). Overview of chemotaxis behavior assays in *Caenorhabditis elegans*. Curr. Protoc..

[69] Rashid S., Wong C., Roy R. (2021). Developmental plasticity and the response to nutrient stress in Caenorhabditis elegans. Dev. Biol..

[70] Ray W.A., Murray K.T., Hall K., Arbogast P.G., Stein C.M. (2012). Azithromycin and the risk of cardiovascular death. N. Engl. J. Med..

[71] Rea S.L., Ventura N., Johnson T.E. (2007). Relationship between mitochondrial electron transport chain dysfunction, development, and life extension in caenorhabditis elegans. PLoS Biol..

[72] Rodriguez-Mozaz S., Vaz-Moreira I., Varela Della Giustina S., Llorca M., Barceló D., Schubert S., Berendonk T.U., Michael-Kordatou I., Fatta-Kassinos D., Martinez J.L., Elpers C., Henriques I., Jaeger T., Schwartz T., Paulshus E., O’Sullivan K., Pärnänen K.M.M., Virta M., Do T.T., Manaia C.M. (2020). Antibiotic residues in final effluents of European wastewater treatment plants and their impact on the aquatic environment. Environ. Int..

[73] Ruszkiewicz J.A., Pinkas A., Miah M.R., Weitz R.L., Lawes M.J.A., Akinyemi A.J., Ijomone O.M., Aschner M. (2018). C. elegans as a model in developmental neurotoxicology. Toxicol. Appl. Pharmacol..

[74] Sam C., Bordoni B. (2024). In *StatPearls*.

[75] Schiffman S.S. (2018). Influence of medications on taste and smell. World J. Otorhinolaryngol. - Head. Neck Surg..

[76] Senta I., Kostanjevecki P., Krizman-Matasic I., Terzic S., Ahel M. (2019). Occurrence and behavior of macrolide antibiotics in municipal wastewater treatment: possible importance of metabolites, synthesis byproducts, and transformation products. Environ. Sci. Technol..

[77] Sharma L., Siedlewicz G., Pazdro K. (2021). The toxic effects of antibiotics on freshwater and marine photosynthetic microorganisms: state of the art. Plants.

[78] Sheikh S., Alvi U., Soliven B., Rezania K. (2021). Drugs that induce or cause deterioration of myasthenia gravis: an update. J. Clin. Med..

[79] Shimizu K., Ashida K., Hotta K., Oka K. (2019). Food deprivation changes chemotaxis behavior in *Caenorhabditis elegans*. Biophys. Phys..

[80] Shukla S., Saxena A., Shukla S.K., Nazir A. (2023). Modulation of neurotransmitter pathways and associated metabolites by systemic silencing of gut genes in C. elegans. Diagnostics.

[81] Singh N.S., Singhal N., Kumar M., Virdi J.S. (2021). High prevalence of drug resistance and class 1 integrons in escherichia coli isolated from river yamuna, india: a serious public health risk. Front. Microbiol..

[82] Singh S., Pal N., Shubham S., Sarma D.K., Verma V., Marotta F., Kumar M. (2023). Polycystic ovary syndrome: etiology, current management, and future therapeutics. J. Clin. Med..

[83] Stuhr N.L., Nhan J.D., Hammerquist A.M., Van Camp B., Reoyo D., Curran S.P. (2022). Rapid lipid quantification in caenorhabditis elegans by oil red O and nile red staining. Bio-Protoc..

[84] Suman S. (2024). Enteric nervous system alterations in inflammatory bowel disease: perspectives and implications. Gastrointest. Disord..

[85] Thongtang N., Sukmawan R., Llanes E.J.B., Lee Z.-V. (2022). Dyslipidemia management for primary prevention of cardiovascular events: best in-clinic practices. Prev. Med. Rep..

[86] Tousova Z., Oswald P., Slobodnik J., Blaha L., Muz M., Hu M., Brack W., Krauss M., Di Paolo C., Tarcai Z., Seiler T.-B., Hollert H., Koprivica S., Ahel M., Schollée J.E., Hollender J., Suter M.J.-F., Hidasi A.O., Schirmer K., Schulze T. (2017). European demonstration program on the effect-based and chemical identification and monitoring of organic pollutants in European surface waters. Sci. Total Environ..

[87] Välitalo P., Kruglova A., Mikola A., Vahala R. (2017). Toxicological impacts of antibiotics on aquatic micro-organisms: A mini-review. Int. J. Hyg. Environ. Health.

[88] Vingskes A.K., Spann N. (2018). The toxicity of a mixture of two antiseptics, triclosan and triclocarban, on reproduction and growth of the nematode Caenorhabditis elegans. Ecotoxicology.

[89] Wang Q., Yu M., Zhang W., Liu B., Zhao Q., Luo X., Xu H., She Y., Zang D., Qiu J., Shen J., Peng Y., Zhao P., Xue L., Chen W., Ma L., Nie X., Shen C., Chen S., Liu Q. (2019). Azithromycin inhibits muscarinic 2 receptor-activated and voltage-activated Ca ^2+^ permeant ion channels and Ca ^2+^ sensitization, relaxing airway smooth muscle contraction. Clin. Exp. Pharmacol. Physiol..

[90] World Health Organization (2012).

[91] Xiong W., Sun Y., Zhang T., Ding X., Li Y., Wang M., Zeng Z. (2015). Antibiotics, antibiotic resistance genes, and bacterial community composition in fresh water aquaculture environment in China. Microb. Ecol..

[92] Yeromina H., Ieromina Z., Fedosov A., Vislous O., Suleiman М, Upyr T., Sych I., Perekhoda L. (2022). A study of surface water pollution with azithromycin in Ukraine. Scr. Sci. Pharm..

[93] Yu J., Chen X., Zhang Y., Cui X., Zhang Z., Guo W., Wang D., Huang S., Chen Y., Hu Y., Zhao C., Qiu J., Li Y., Meng M., Guo M., Shen F., Zhang M., Zhou B., Gu X., Xu L. (2022). Antibiotic azithromycin inhibits brown/beige fat functionality and promotes obesity in human and rodents. Theranostics.

[94] Yu Z., Wang L., Li G., Zhang J. (2023). Reproductive influences of erythromycin and sulfamethoxazole on caenorhabditis elegans over generations mediated by lipid metabolism. Chem. Res. Chin. Univ..

[95] Yu Z., Zhang J., Yin D. (2016). Multigenerational effects of heavy metals on feeding, growth, initial reproduction and antioxidants in caenorhabditis elegans. PLOS ONE.

[96] Zaman Huri H., Lim L.P., Lim S.K. (2015). Glycemic control and antidiabetic drugs in type 2 diabetes mellitus patients with renal complications. Drug Des., Dev. Ther..

[97] Zhang M.-Q., Wu G.-Z., Zhang J.-P., Hu C.-Q. (2023). The comparative analysis of gastrointestinal toxicity of azithromycin and 3′-decladinosyl azithromycin on zebrafish larvae. Toxicol. Appl. Pharmacol..

[98] Zhang Y., Xiu W., Yan M., Guo X., Ni Z., Gu J., Tang T., Liu F. (2023). Adverse effects of sulfamethoxazole on locomotor behavior and lipid metabolism by inhibiting acetylcholinesterase and lipase in *Daphnia magna*. Sci. Total Environ..

[99] Zheng Y., Yu Z., Zhang J. (2022). Multi-generational effects of enrofloxacin on lifespan and reproduction of Caenorhabditis elegans with SKN-1-mediated antioxidant responses and lipid metabolism disturbances. Sci. Total Environ..

